# The Value of Opium in Inflammation, Especially When Arising from Severe Gun-Shot Wounds

**Published:** 1865-02

**Authors:** Joel Hall

**Affiliations:** P. A. C. S.


ART. III.—*The Value of Opium in Inflammation, especially when arising from severe Gun-shot wounds.* By JOEL HALL, P. A. C. S.

The history of this case may be uninteresting in other respects, except in a statistical point of view. The mortality which has hitherto attended penetrating wounds of the abdomen should prompt every observer to report the result of such cases, particularly noting the circumstances, symptoms, &c., of those cases terminating in recovery.

*History.*—Sergeant W. T. Price, company "H," 15th Tennessee regiment, aged 23, of good constitution and health, a farmer by occupation previous to entering the service, was wounded on the evening of November 1st, 1864, by the accidental discharge of an Enfield rifle, at very short range.—The ball entered the abdomen just below the sup. spin. proc. of right ilium, anterior to the bone, passing through transversely and escaping through the left inguinal region without injuring the bladder or intestines.

I reached the patient five minutes after receipt of injury, and introduced my finger into the wound to ascertain the extent of injury. After having satisfied myself that the cavity of the abdomen had been penetrated, a catheter was passed. A small quantity of urine issued, not exceeding two or three fluid ounces, which was not mingled with blood. Patient stated that he had voided urine a few minutes before. An æenema of soap and water was next administered, which was soon cast off, showing no trace of intestinal injury. Shock very decided. Administered one fluid ounce of whiskey and one grain of opium. No hospital being near, placed him upon a litter and conveyed him to Tuscumbia, Ala., two miles distant, and placed him confortably in bed. Applied a single dressing to the wound. Administered one grain of opium at 9 o'clock, P. M., and another at 3 o'clock, A. M.

November 2d.—Rested well last night. Pulse eighty this morning. Ordered half a grain of opium every three hours during the day. Six o'clock, P. M., no change. Ordered one grain of opium to be given at 9 o'clock, and another a 4 o'clock, A. M. Voided urine late this evening. Bowels quiet.

November 3d.—Slept well. Bowels still quiet. No tympanites existing. Voided urine without difficulty this morning. Pulse eighty-five. Treatment continued.

November 4th.—Bowels have not moved yet. Pulse ninety. No symptoms indicating extensive peritonitis. Wound beginning to suppurate slightly. Ordered a little rice water and continuation of treatment.

November 5th.—Slept well last night. Pulse eighty-five this morning. Voided urine during the night. In other respects, same as yesterday. Ordered half a grain of opium every four hours during the day.

November 6th.—No change. Treatment continued.

November 7th.—Rested well last night. Bowels a little flatulent. Pulse eighty-five. Appetite good. Ordered an ounce of boiled sweet milk every six hours; half a grain of opium every five hours during the day; cold water dressing to wounds, which has been kept up from the beginning.

November 8th.—Same as yesterday. Treatment continued.

November 9th.—Pulse eighty. Appetite good. In other respects, no change.

November 10th.—Bowels moved twice last night. Pulse seventy-five. Appetite good. Patient doing well. Diet improved. Treatment continued.

November 11th.—Wound suppurating freely. Pulse seventy-five. Appetite continues good. Bowels moved once in last twenty-four hours. Ordered half a grain of opium every six hours. Wound to be dressed as heretofore and kept clean.

November 12th.—No change.

November 13th.—Improving rapidly. Opium to be discontinued, except to procure sleep at night.

Here my notes cease. The corps moved from Tuscumbia across the Tennessee river, to Florence, and the patient was placed in charge of a local physician. Although the case has not yet recovered, I feel that nothing is hazarded in expressing the belief that he will soon be well. Whatever is known of the future result of the case, will be borne upon the monthly report of "Surgical Cases."

